# Recovery-Oriented Systems of Care: A Perspective on the Past, Present, and Future

**DOI:** 10.35946/arcr.v41.1.09

**Published:** 2021-07-22

**Authors:** Larry Davidson, Michael Rowe, Paul DiLeo, Chyrell Bellamy, Miriam Delphin-Rittmon

**Affiliations:** 1 Program for Recovery and Community Health, Department of Psychiatry, Yale University School of Medicine, New Haven, Connecticut; 2 Connecticut Department of Mental Health and Addiction Services, Hartford, Connecticut; 3 Yale University School of Medicine, New Haven, Connecticut

**Keywords:** mental health recovery, substance use recovery, recovery-oriented care, behavioral health, recovering citizenship, recovery-oriented system of care, alcohol

## Abstract

This paper provides a perspective on the recent concept of recovery-oriented systems of care with respect to its origins in the past and its status in the present, prior to considering directions in which such systems might move in the future. Although influential in practice, this concept has yet to be evaluated empirically and has not been the object of a review. Recovery-oriented systems of care emerged from the efforts of persons with mental health and/or substance use disorders who advocated for services to go beyond the reduction of symptoms and substance use to promote a life in the community. Subsequent efforts were made to delineate the nature and principles of such services and those required of a system of such care. Coincident with the U.S. Substance Abuse and Mental Health Services Administration dropping reference to behavioral health in its revised definition of recovery, confusions and limitations began to emerge. Recovery appeared to refer more to a process of self-actualization for which an individual is responsible than to a process of healing from the effects of a behavioral health condition and associated stigma. In response, some systems are aiming to address social determinants of behavioral health conditions that transcend the scope of the individual and to develop a citizenship-oriented approach to promote community inclusion.

This perspective focuses on the relatively recent topic of recovery-oriented systems of care; although influential in practice, this concept has yet to be evaluated empirically and thus cannot yet be the object of a review. In lieu of such evidence, this article offers one perspective on the origins of this concept and its present status prior to considering possible directions in which such systems might move in the future. In the process, areas in which research is especially needed are highlighted to evaluate the utility of this concept in meeting its stated aim of moving behavioral health systems of care beyond an acute care model to better meet the needs of persons with prolonged mental illness and/or substance use disorder (SUD). Throughout this perspective, “substance use” refers to both alcohol use and other drug use.

This story begins with the decade between 2000 and 2010, which saw a flurry of activity at the federal level in the United States focused on defining what was then the relatively new concept of recovery in both mental health and substance use. Although other concepts of recovery may be as old as the treatment and study of mental health (e.g., Phillippe Pinel and moral treatment)^1^ and SUD (e.g., 12-step tradition),^2^ the term was given new meanings in the 1980s and 1990s through the consumer/survivor movement in mental health^3^ and the new recovery advocacy movement in substance use.^4^ These new definitions were then operationalized in terms of their implications for transforming mental health and SUD services to promote these new forms of recovery. At least two central arguments for the shift to recovery and recovery-oriented care were consistent across the mental health and substance use divide.

First, there was a growing recognition that although full (“clinical”) recovery was possible following an acute episode of a mental or substance use disorder for some people, a more personal sense of recovery—involving a process of learning how to manage daily life in the presence of, or within the limitations imposed by, an ongoing disorder—was required and appropriate for others. Second, there was a parallel recognition that mental health and substance use services were primarily oriented to providing acute care that targeted, and hopefully lessened, signs and symptoms of mental disorder and substance use while paying considerably less attention to promoting functioning and living a full, meaningful life in the community of one’s choice.

This perspective considers the implications of these two arguments for transforming mental health and substance use services under the broad vision of recovery-oriented systems of care, which has since been developed with support from the Substance Abuse and Mental Health Services Administration (SAMHSA). In addition to describing the initial steps taken during the 2000–2010 decade, this article considers the current status of additional efforts made between 2010 and 2020, prior to offering possible strategies to overcome some of the confusions and limitations that have been identified within the context of efforts to implement this ambitious vision. In the absence of empirical studies of this relatively new way of organizing behavioral health care, this perspective uses as a case study the evolution of mental health and substance use services in Connecticut, which was the first state in the country to envision and attempt to achieve a recovery-oriented system of care that both integrates mental health and substance use services and reorients them to promoting the new senses of recovery articulated by the recovery community itself.^5^,^6^ Given that recovery-oriented systems of care emphasize prevention, health promotion, and outreach to, and inclusion of, persons with multiple conditions, no recovery-oriented system of care to date has specifically targeted persons solely with alcohol use disorder.

## THE PAST: 2000–2010

The concept of recovery has been pushed to the forefront of behavioral health policy and practice in the United States (and elsewhere) over the last 3 decades largely through the advocacy efforts of people with behavioral health disorders rather than through advances in the effectiveness of new psychiatric medications or an accumulating body of research on clinical improvements or positive outcomes in the treatment of SUD.^7^ Before it referred to innovations in practice, recovery referred to the right of people with behavioral health conditions to “live, work, learn, and participate fully in the community.”^8^ Based most recently on the Americans with Disabilities Act of 1990^9^—but grounded in 30 years of consistent federal law preceding it (e.g., the Rehabilitation Act of 1973)^10^—this right cannot be made contingent on improvements in the person’s clinical or functional status, nor can it be delayed indefinitely based on a system’s lack of resources to support community tenure. Persons with behavioral health disorders have a right to live in the community alongside their peers and to participate in the treatment and rehabilitative interventions and make use of the community supports they need to manage their behavioral health conditions and pursue their own life goals. The challenge for a recovery-oriented system of care is to carry out this work in the most efficient and effective, and the least coercive and restrictive, manner possible, respecting the dignity and autonomy of clients while ensuring the safety and well-being of the broader community.

To guide these efforts, SAMHSA first held consensus development conferences separately for the mental health and substance use communities. The agency later brought them together around 2010 to come up with an integrated definition, reviewed below. It is worth citing the initial definitions, however, to get a sense of the direction in which SAMHSA was moving during the first decade of the 2000s. As defined by the 2004 National Consensus Statement on Mental Health Recovery, “Mental health recovery is a journey of healing and transformation enabling a person with a mental health problem to live a meaningful life in a community of his or her choice while striving to achieve his or her full potential.”^11^ “Recovery from alcohol and drug problems,” on the other hand, was defined in a 2005 SAMHSA consensus statement as “a process of change through which an individual achieves abstinence and improved health, wellness, and quality of life.”^12^ These definitions can be seen for the most part as compatible, the only real difference being that one focuses on mental health problems and the other on alcohol and drug problems.

While it is clear from these definitions that this form of recovery is viewed as a process in which the person must be actively engaged, they hold implications for the nature of behavioral health treatment and supports as well. In other words, although a person needs to engage in their own recovery, making use of recovery-oriented services and supports can be one element of one’s personal recovery efforts. This notion was first introduced in 2000, when Anthony published an important paper, “A recovery-oriented service system: Setting some system level standards.”^13^ This article laid out the argument for what standards should be used in evaluating treatments and community supports as to their recovery-orientation—that is, the degree to which the services and supports offered are aimed at promoting this new vision of recovery as the person’s living a meaningful life, achieving one’s full potential, and improving one’s health and wellness in the presence of a behavioral health problem. Building on these efforts, in 2002 Connecticut became the first state behavioral health authority to adopt a commissioner’s policy on promoting a recovery-oriented system of care. In this early stage, such a system was defined as one “that identifies and builds on each individual’s assets, strengths, and areas of health and competence to support each person in achieving a sense of mastery over mental illness and/or SUD while regaining his or her life and a meaningful, constructive sense of membership in the broader community.”^14^

Expanding upon these and similar efforts around the country, in 2010, SAMHSA came out with its own definition of a recovery-oriented system of care: “a coordinated network of community-based services and supports that is person-centered and builds on the strengths and resiliencies of individuals, families, and communities to achieve improved health, wellness, and quality of life for those with or at risk for mental health and substance use problems.”^15^ SAMHSA’s vision of a recovery-oriented system of care encompasses a menu of individualized, person-centered, and strength-based services within a self-defined network. This menu includes clinical services and alternative therapies (such as acupuncture and meditation) as well as recovery support services. Recovery support services include peer recovery coaching and other forms of peer support, peer-run programs, recovery community centers, employment and educational assistance, social and family support, childcare, care management, and housing support. It also provides individuals and families with more options with which to make informed decisions regarding their care; is designed to be accessible, welcoming, and easy to navigate; involves people in recovery, their families and allies, and the broader community to continually improve access to and quality of services; and supports the premise that there are many pathways to recovery.

Finally, recovery-oriented systems of care have been the focus of various technical assistance resources issued by SAMHSA, in which such systems are described as adhering to a list of principles and as serving specific functions.^15^–^18^ But what does such a system actually look like? Based on the stages of change model first introduced into treatment of substance use, the overarching principle for design of this system and its various components is that people should be able to access effective and responsive services and supports regardless of where they are in the process of recovery from SUD, mental illness, or both combined. Realizing that substance use and mental health disorders frequently co-occur, this model further allows for a person to be in different stages with respect to each of the conditions they may have. Most importantly, being unaware of, or choosing not to accept having, a behavioral health condition is to be viewed as a point of departure for treatment, rehabilitation, and support efforts as opposed to being viewed as cause for discharge from care. Based also on the input of people who are in recovery, this model places central emphasis on the role of recovery support services, including services provided by peers, at each point along the continuum of care.

It could be argued that within a recovery-oriented system of care, all services should be supportive of recovery. The term “recovery support services” has been used, however, to refer more specifically to a subgroup of interventions—particularly those that focus on enhancing a person’s abilities and resources, or recovery capital, to manage their own behavioral health condition(s) and/or to increase their participation in the community activities of their choice.^18^,^19^ Importantly, these services and resources are to be offered to persons entering recovery prior to (as well as during and after) any expectations that they accept and benefit from active treatment rather than being reserved as rewards for doing so. People may need a basic amount of recovery capital to be able to make effective use of such treatments, whether medication or psychosocial. Finally, recovery support services are often provided by people who are in recovery themselves, but do not need to be exclusively so. Being a relatively recent development and given their central role in knitting such systems of care together, recovery support services will be an especially important topic for future research.

**Figure 1:**
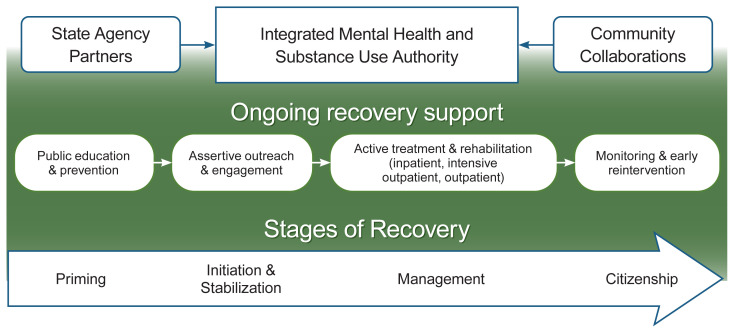
Recovery-oriented system of care An integrated mental health and substance use authority provides care throughout the stages of recovery, beginning with public education, prevention, and mental health promotion. For those who do not seek care on their own, assertive outreach and engagement efforts provide outreach to people in distress or need, wherever they are. Active treatment and rehabilitation are supported with recovery support services, which helps to increase service engagement and effectiveness. Ongoing monitoring and early reintervention are provided as needed. State agencies and community collaborators act as partners to support the efforts of the integrated behavioral health authority.

As shown in Figure 1, these services and supports also can be used during various stages of recovery and are conceptualized with a recovery management model, in which they assertively strive, according to White and Kelly, to “enhance early pre-recovery engagement, recovery initiation, long-term recovery maintenance, and the quality of personal/family life in long-term recovery.”^20^ The stages span from recovery priming (i.e., having experiences that prepare the person to make the decision to pursue recovery), to recovery initiation and stabilization, to recovery management and, finally, recovering one’s full citizenship as a valued member of one’s community. This model has been developed based on the arguments cited in the introduction: that is, that many substance use disorders, like many mental illnesses, are prolonged rather than acute in nature, calling for services and supports to be offered to persons over longer periods of time and consistent with where they may be in recovery at the time. According to White and Kelly,^20^ this model thus involves focused attention at several points along a continuum of care and across levels and components of the system that is managed by an overall integrated mental health and substance use authority (whether at the local, regional, or state level), including the following: (1) public education and prevention; (2) continuity of contact over a sustained period of time; (3) patient/family education and empowerment to promote self-management of the condition (including mobilization of family resources); (4) access to the latest advances in medication-assisted treatment; (5) access to peer-based recovery support groups and advocacy organizations; and (6) sustained monitoring (checkups), recovery coaching, and when needed, early re-intervention.

As can be seen in this figure, the continuum of care begins with public education, prevention, and mental health promotion. Then, for those who do not seek care on their own, assertive outreach and engagement efforts can take place anywhere—from the streets to faith communities, college campuses, and workplace settings—reaching out to people in distress or need wherever they might be found. At this point, recovery support services can be introduced to overcome barriers to access to care, to offer environments supportive of recovery, or to help to increase the person’s recovery capital so that treatment, when accessed, can be fully effective. These kinds of recovery support should be available to persons in recovery throughout the remainder of their journey, either in different forms depending on the stage of change (e.g., case management until the person has established a firm foundation for recovery) or in a consistent form depending on the person’s choice (e.g., 12-step group, recovery community center). Following various forms of active treatment (e.g., detox/inpatient, intensive outpatient, outpatient), support is available for ongoing monitoring (e.g., wellness checkups) and early reintervention as needed. This continuum of care is developed in collaboration with a wide range of stakeholder partners, including education and faith community leaders, police and criminal justice representatives, business owners and other employers, family members and allies, and, perhaps most important, representatives of the recovery community itself.

## THE PRESENT: 2010–2020

Such was the vision put forth beginning around 2000 as new meanings of recovery began to take hold, along with implications for transforming services, supports, and systems of care. And much progress has been made in the past 20 years in bringing this vision to life. Public education, including school-based efforts, have begun to address the roles of stigma and discrimination as barriers to access to care and to recovery, including the role of medications in the treatment of both mental illness and SUD. Inroads have been made into faith communities and onto college campuses to promote behavioral health and to increase access to needed services and supports. Increasing numbers of highly visible role models of recovery have disclosed their own struggles with mental illness and/or SUD and encouraged their followers and fans to know that help is available and how to ask for it. An expanding array of recovery support services are being offered and are beginning to be shown useful in increasing access to and the effectiveness of care.^21^–^24^ So, other than continuing to follow this blueprint in building systems of recovery-oriented care, what remains to be done?

Unfortunately, over the last 10 years, confusion has arisen and limitations have been identified related to these notions of recovery and recovery-oriented care, threatening further progress toward a recovery orientation and with the potential, perhaps, to turn the clock backward. Although this confusion and these limitations do not stem directly from the more recent SAMHSA definition of recovery, they nonetheless seem to be best captured in the differences between the initial definitions cited above and the integrated version issued as a working definition in 2012. Hoping to integrate mental health and substance use services under a single umbrella, SAMHSA initiated another consensus development process in 2010 that involved representatives from both recovery communities and other stakeholders; this resulted in the following working definition of recovery from mental illness and/or SUD: Recovery is “a process of change through which individuals improve their health and wellness, live a self-directed life, and strive to reach their full potential.”^25^ Possibly due to pushback from some mental health and substance use recovery advocates who opposed the idea of behavioral health conditions being framed as disorders, what is conspicuously absent from this definition is what the person is recovering from. This definition appears to apply equally well to those without, as well as to those with, a mental illness or SUD. In this sense, the definition could apply equally well to everyone while saying nothing specific about anyone.

The advocates’ point is well-taken and important, however. Persons with what has been described as mental illness or SUD are first and foremost, and most fundamentally, human beings just like everyone else. But if they remain human beings just like everyone else in all respects, then they lose their justification for laying claim to funding for behavioral health services and supports. If all that a person is doing is engaging in “a process of change” through which they are hoping to improve their “health and wellness, live a self-directed life, and strive to reach” their “full potential,” then society has no obligation to provide them with different types of support or any more support than anyone else. In addition, this process is not only self-directed, but also appears to be entirely up to the individual. It appears to be their responsibility, and theirs only, to live their self-directed life as they wish. If they encounter difficulties in doing so, they are entirely responsible for managing these challenges, and they have no fundamental right to claim any relief or intervention from anyone else.

How different this is from the framing of the Americans with Disabilities Act of 1990, which ushered in the recovery movement, in which mental illness and SUD were considered to be disabilities that entitled persons to request and receive reasonable accommodations and community supports necessary to live as full a life as desired alongside their peers without disabilities in the community of their choice.^26^ That too was the result of considerable advocacy efforts. At least one major confusion and one major limitation have emerged from the shift from a disability model, in which services and supports are essential to ensuring persons’ rights to community inclusion, to what may be called a self-actualization model, in which everyone could be recovering from something and so no one has a particular right to anything. This perspective addresses each of these in turn.

First, in the self-actualization model, there is the perception, or the implication, that recovery is the sole responsibility of the individual. Although people certainly play a central role in their own recovery, neither the person nor their recovery occurs in a vacuum and most often benefits from a supportive social context inclusive of accessible services and supports. Viewing recovery as solely the person’s own responsibility delegitimizes the important roles that services and supports can play in lessening the suffering, burdens, and intrusions of the disorders and in promoting and enabling the degree of functioning required to lead a satisfying and meaningful life in one’s community. Most often, such a confusion of viewing personal recovery as a personal responsibility has been used as justification for drawing arbitrary limits on the use of, or denying access entirely to, behavioral health services and supports to persons in need.^27^ Either people claiming to be “in recovery” are considered too well to require care any longer or their ongoing challenges are viewed as requiring a different type of service than those provided based on medical necessity, thus garnering fewer resources. That is, if recovery is simply and solely an individual’s journey to “reach their full potential,” then “Good luck with that journey,” the behavioral health system need only provide them with minimal, if any, support.

Second, understanding recovery as a personal journey for which the individual is largely responsible has the added byproduct of leading to a discounting of all those forces beyond the individual that are known to influence the onset, course, and outcomes of mental illness and SUD. These social determinants of mental illness and SUD include poverty, unstable housing, prolonged involuntary unemployment, social exclusion and isolation, and various forms of stigma and discrimination based on health status, gender, race and ethnicity, sexual orientation, religious and cultural orientation, and other markers of difference.^28^–^31^ Understanding recovery as an individual’s responsibility may draw attention away from the array of social conditions and collective resources needed for even the possibility of recovery (i.e., it is extremely difficult to recover without having a home, a family or friends, and an income). This use of recovery as a diversion of attention away from social, political, economic, and cultural factors has become such a serious concern among some earlier recovery proponents that articles have begun to appear with titles such as “Uses and Abuses of Recovery,”^32^ and coalitions have begun to form to combat the political use of recovery as an excuse for preserving current inequities. One such coalition, Recovery in the Bin, clearly expresses this concern on its website as follows: “We recognise that the growing development of [mental health] ‘Recovery’ . . . has been corrupted by neoliberalism and capitalism is the crisis! Some of us will never feel ‘Recovered’ living under these intolerable inhumane social . . . and economic conditions, such as poor housing, poverty, stigma, racism, sexism, unreasonable work expectations, and countless other barriers.”^33^

The confusion of personal recovery with (solely) personal responsibility appears to have limited the concept of recovery to an artificially decontextualized personal sphere that is somehow immune to the social determinants of mental health and substance use. If so, what might the future hold for still developing recovery-oriented systems of care? Although research is still sorely needed on this topic, a case study of Connecticut’s experience sheds some light on an answer to this question.

## A POSSIBLE FUTURE

In Connecticut, in order to address and overcome these issues, this perspective found it necessary to incorporate an explicit focus on the array of social, economic, political, and cultural determinants of mental health and substance use and an emphasis on community inclusion and community life as a collective phenomenon into the state’s recovery transformation work.^34^–^40^ Doing so has required returning to the consumer/survivor and new recovery advocacy movements, which themselves are rooted, in part, in the civil rights movement of the 1950s and 1960s and the independent living and disability rights movement of the 1970s.^41^ It was these movements, and the legislation inspired by them (e.g., the Rehabilitation Act of 1973, the Americans with Disabilities Act of 1990), that established the rights of persons with functionally disabling conditions (based on a medical assessment of functional impairment) to be provided not only with medical care for their health condition but also with the community supports needed to be able to live full and dignified lives in the communities of their choice. Were mental illness and SUD not recognized as legitimate health conditions, it is difficult to see how funding such supports could be justified. This may mean that some tensions between a state mental health and substance use authority and various advocacy communities are inevitable to some degree, although hopefully there remains much common ground to be found and put to good use.

In addition to returning to its roots in a disability rights paradigm,^7^,^28^ this national shift in the direction of transformation is grounded in more than 20 years of research and scholarship related to the concept of “citizenship.”^36^,^37^ Although this concept has begun to gain traction in the mental health field over the last decade,^38^–^41^ it is relatively new and less widely known than the concept of recovery. A rich and important topic for research in its own right, the concept of citizenship also has been especially effective as a counterbalance to the overemphasis on the individual nature of recovery discussed above. It is in this spirit—as drawing attention both to the social determinants of behavioral health and to the collective nature of community life—that the state of Connecticut’s Department of Mental Health and Addiction Services has proposed a few modifications to the model of a recovery-oriented system of care under the rubric of “recovering citizenship.”^42^

Rowe has defined citizenship in the technical sense as a person’s strong connection to the rights, responsibilities, roles, resources, and relationships (the 5 Rs) that a democratic society makes available to its members through public and social institutions, the associational life of voluntary organizations such as faith communities and neighborhood organizations, and social networks and everyday social interactions. It also involves a sense of belonging in a person’s own community that must be validated by others’ recognition of their value as a member of society.^41^ This concept thus builds on the aspect of “a life in the community” that has been core to the definition of personal recovery, spelling out concretely, and helpfully, what such a life is made up of in terms that are not limited to the individual. It recognizes that a person cannot effectively belong to a community unless they are treated as such by others, and that membership in a community comes with certain entitlements and obligations. To recover (or to develop for the first time) the sense of being a full citizen, the person must have certain rights (e.g., the right to community inclusion) and resources (e.g., a home, an income) and be able to take on certain roles and responsibilities (e.g., neighbor, voter) while having meaningful relationships with others that offer the person a sense of belonging. Once spelled out in this way, it becomes obvious how recovery involves more than an individual’s own efforts. A person cannot will themselves to have a sense of belonging to a community; that sense must be conveyed by how others treat the person. Recovery happens in a social context, and that context matters a great deal.

What implications does this emphasis have for our recovery-oriented systems of care? In the model depicted in Figure 1, both state agencies and community collaborators must act as partners in expanding the scope of the behavioral health system to include the full community of people it serves. Although housing may have been recognized decades ago as an essential cornerstone of recovery, similar steps now need to be taken with respect to other components of community life including education, employment, finances, and social, leisure, and artistic pursuits. Along with partnering between the behavioral health authority and the state, county, or city departments that oversee these aspects of community life, inroads can be made into the voluntary sector, civic institutions (e.g., libraries), faith communities, and neighborhood organizations. Just as people with SUD and/or mental illness need to take steps in their own recovery that require courage and risk of failure, communities also need to take steps to welcome, include, and support those with behavioral health disorders. Systems of care oriented toward recovering citizenship recognize the importance of working collaboratively with an array of community leaders and institutions to cultivate opportunities for win-win strategies in which people with disabilities make valuable and valued contributions to their communities that benefit everyone. Giving back in this way has long been a core component of the 12-step tradition in substance use recovery. Forging pathways for people in recovery to have opportunities to do so can be a core component of behavioral health systems more broadly, and empirical studies will be needed to show the influence of this component on health outcomes.

## CONCLUSION

Behavioral health conditions continue to be among the most poorly understood and most stigmatized conditions in the United States. As a result, persons affected by these conditions often face discrimination in how they are viewed and treated by others in numerous arenas, including where they will live, whether they will complete their education or be employed, and which opportunities they will have for participating in community life. To the degree to which the recovery movement remains rooted in a human rights movement, addressing and eliminating these forms of discrimination must be considered a pressing and ongoing priority for systems of care. Doing so is identified as a core function of recovery-oriented practice because little progress will be made either in system transformation or in the social inclusion of persons with behavioral health conditions until they are seen as full citizens of the society to which they belong, with all of the rights and responsibilities associated with membership.

As long as stigma and discrimination continue to exist, persons with behavioral health needs are discouraged from seeking care, but that is not all. They also are being denied the very resources and supports they need to enter into and sustain recovery, such as hope, a sense of meaning and purpose in life, a sense of agency and efficacy, a sense of self-worth, and confidence in their own ability to make good choices. Without these capacities, it becomes extremely difficult for people to voluntarily choose treatment or to take up and persist in the challenging work of recovery. And restoration of these capacities, as well as other forms of recovery capital, cannot be postponed until the person no longer shows any signs or symptoms of behavioral health difficulties.

In this respect, it is important to note that citizenship, including the right to social inclusion, is considered to be a foundation for recovery rather than to be viewed as one of its rewards.^43^ The task of addressing stigma and discrimination comes first, rather than last, because all people have the right to be treated with dignity and respect, regardless of their behavioral health condition or status. In the past, many of the practices of the behavioral health system, as well as of society at large, conveyed the message that people were not welcome in the community as long as they were experiencing behavioral health difficulties. They might be accepted back once recovered (e.g., on release from residential treatment or the hospital), but recovery was viewed as largely out of reach. It has been this combination of stigma and hopeless attitudes that has discouraged many people from seeking care and led others to believe that recovery was not possible for them. Organizations oriented toward recovering citizenship play a key role in shifting the culture both of the behavioral health system and of the broader society in the positive direction of embracing the reality of recovery and valuing the contributions that are made by the recovery community.

## References

[1] Davidson L, Rakfeldt J, Strauss JS (2010). The Roots of the Recovery Movement in Psychiatry: Lessons Learned.

[2] Davidson L, White W, Sells D (2010). Enabling or engaging? The role of recovery support services in addiction recovery. Alcohol Treat Q.

[3] Chamberlin J (1978). On Our Own: Patient Controlled Alternatives to the Mental Health System.

[4] Davidson L, White W (2007). The concept of recovery as an organizing principle for integrating mental health and addiction services. J Behav Health Serv Res.

[5] Davidson L, Tondora J, O’Connell MJ (2007). Creating a recovery-oriented system of behavioral health care: Moving from concept to reality. Psychiatr Rehabil J.

[6] Substance Abuse and Mental Health Services Administration (SAMHSA) (2009). Guiding Principles and Elements of Recovery-Oriented Systems of Care: What Do We Know from the Research?.

[7] Davidson L (2006). What happened to civil rights?. Psychiatr Rehab J.

[8] United States President’s New Freedom Commission on Mental Health (2003). Achieving the Promise: Transforming Mental Health Care in America.

[b9-arcr-41-1-9] 9Americans With Disabilities Act of 1990, 42 U.S.C. § 12101 et seq. (1990). https://uscode.house.gov/view.xhtml?req=granuleid%3AUSC-prelim-title42-chapter126&edition=prelim

[b10-arcr-41-1-9] 10Rehabilitation Act of 1973, 29 U.S.C. § 701 et seq. https://www.govinfo.gov/app/details/USCODE-2015-title29/USCODE-2015-title29-chap16-other-sec701

[11] SAMHSA (2004). National Consensus Statement on Mental Health Recovery.

[12] SAMHSA (2005). National Consensus Statement on Recovery.

[13] Anthony WA (2000). A recovery-oriented service system: Setting some system-level standards. Psychiatr Rehabil J.

[14] Connecticut Department of Mental Health and Addiction Services (2002). Commissioner’s Policy Statement No. 83: Promoting a Recovery-Oriented System of Care.

[15] SAMHSA (2010). Recovery-Oriented System of Care (ROSC) Resource Guide. Working draft.

[16] Sheedy CK, Whitter M (2009). Guiding Principles and Elements of Recovery-Oriented Systems of Care: What Do We Know From the Research?.

[17] Whitter M, Hillman DJ, Powers P (2010). Recovery-Oriented Systems of Care (ROSC) Resource Guide.

[18] Kaplan L (2008). The Role of Recovery Support Services in Recovery-Oriented Systems of Care.

[19] White WL (2008). Recovery Management and Recovery-Oriented Systems of Care: Scientific Rationale and Promising Practices.

[20] White WL, Kelly JF, Kelly JF, White WL (2011). Recovery management: What if we really believed that addiction was a chronic disorder?. Current Clinical Psychiatry. Addiction Recovery Management: Theory, Research and Practice.

[21] Laudet AB, Humphreys K (2013). Promoting recovery in an evolving policy context: What do we know and what do we need to know about recovery support services?. J Subst Abuse Treat.

[22] Bassuk EL, Hanson J, Greene RN (2016). Peer-delivered recovery support services for addictions in the United States: A systematic review. J Subst Abuse Treat.

[23] Reif S, Braude L, Lyman DR (2014). Peer recovery support for individuals with substance use disorders: Assessing the evidence. Psychiatr Serv.

[24] White WL (2009). Peer-Based Addiction Recovery Support: History, Theory, Practice and Scientific Evaluation.

[25] SAMHSA (2012). SAMHSA’s working definition of recovery.

[26] Davidson L (2016). The recovery movement: Implications for mental health care and enabling people to participate fully in life. Health Affairs.

[27] Braslow JT (2013). The manufacture of recovery. Annu Rev Clin Psychol.

[28] Davidson L (2018). Is there a future for recovery?. World Association for Psychosocial Rehabilitation Bulletin.

[29] Compton MT, Shim RS (2015). The social determinants of mental health. Focus.

[30] Harper D, Speed E, Speed E, Moncrieff J, Rapley M (2014). Uncovering recovery: The resistible rise of recovery and resilience. De-Medicalizing Misery, II: Society, Politics, and the Mental Health Industry.

[31] Morrow M, LeFrançois BA, Menzies R, Reaume G (2013). Recovery: Progressive paradigm or neoliberal smokescreen. Mad Matters: A Critical Reader in Canadian Mad Studies.

[32] Slade M, Amering M, Farkas M (2014). Uses and abuses of recovery: Implementing recovery-oriented practices in mental health systems. World Psychiatry.

[33] Recovery in the Bin.

[34] Quinn N, Bromage B, Rowe M (2019). Collective citizenship: From citizenship and mental health to citizenship and solidarity. Soc Policy Admin.

[35] Fleischer DZ, Zames F (2001). The Disability Rights Movement:From Charity to Confrontation.

[36] Rowe M (1999). Crossing the Border: Encounters Between Homeless People and Outreach Workers.

[37] Rowe M (2014). Citizenship and Mental Health.

[38] Pelletier JF, Davidson L, Roelant JL (2009). Citizenship and recovery for everyone: A global model of public mental health. Int J Ment Health Promot.

[39] Pelletier JF, Corbière M, Lecomte T (2015). Citizenship and recovery: Two intertwined concepts for civic-recovery. BMC Psychiatry.

[40] Eiroa-Orosa FJ, Rowe M (2017). Taking the concept of citizenship in mental health across countries: Reflections on transferring principles and practice to different sociocultural contexts. Front Psychol.

[41] Rowe M, Ponce AN, Goldman HH, Frank RG, Morrissey JP (2019). How shall we promote citizenship and social participation?. Handbook of U.S. Mental Health Policy.

[42] Rowe M, Davidson L (2016). Recovering citizenship. Isr J Psychiatry Relat Sci.

[43] Davidson L, Tondora J, O’Connell MJ (2009). A Practical Guide to Recovery-Oriented Practice: Tools for Transforming Mental Health Care.

